# Correlation of cooking time with water absorption and changes in relative density during boiling of cassava roots

**DOI:** 10.1111/ijfs.14769

**Published:** 2020-08-30

**Authors:** Thierry Tran, Xiaofei Zhang, Hernan Ceballos, Jhon L. Moreno, Jorge Luna, Andrés Escobar, Nelson Morante, John Belalcazar, Luis A. Becerra, Dominique Dufour

**Affiliations:** ^1^ CGIAR Research Program on Roots Tubers and Bananas (RTB) The Alliance of Bioversity International and the International Center for Tropical Agriculture (CIAT) Cali Colombia; ^2^ French Agricultural Research Centre for International Development (CIRAD) UMR Qualisud Cali Colombia; ^3^ Qualisud Univ Montpellier CIRAD Montpellier SupAgro Univ d'Avignon Univ de La Réunion Montpellier France; ^4^ CIRAD UMR QUALISUD Montpellier France

**Keywords:** Cooking quality, relative root density, dry matter content, high‐throughput phenotyping (HTPP), cassava quality, breeding, consumer preferences

## Abstract

Consumers prefer cassava roots that cook quickly during boiling. Current methods to evaluate cooking time (CT) are slow and labour‐intensive. This article describes improved protocols for assessing CT in roots. We evaluated CT in 36 genotypes monthly at 8–11 months after planting. CT showed differences for plant age at harvest and among genotypes. During boiling, roots absorbed water (WAB) and thus reduced their relative density (DEN). We classified three groups of genotypes with increasing CT (≤25 min, 25–40 min and >40 min), associated with decreasing WAB, respectively, 15.3 ± 3.1, 10.7 ± 1.7 and 4.9 ± 3.8% of initial root weight. A similar trend was observed for changes in DEN (46.3 ± 9.8, 54.5 ± 11.1 and 75.9 ± 6.9% of initial DEN, respectively). The highest correlations between WAB and DEN with CT (*r* ^2^ > 0.6) were found at 30‐min boiling. These alternative protocols facilitate screening large numbers of cassava genotypes for CT.

## Graphical abstract

Genetic differences in boiled cassava roots. The photographs on the right illustrate contrasting examples of short and long cooking time. The appearance of friable roots (a trait required by consumers) changes during boiling. Roots with good cooking quality absorb water abundantly and quickly during boiling. As a result, their relative density is reduced. The graph in the top illustrates water absorption during boiling expressed as percentage of the initial weight of root samples from selected genotypes. The data are averages of four harvests at different ages of the plant (from 8 to 11 months after planting). The graph at the bottom presents the change of weight in water (expressed as percentage of the initial weight) during boiling roots from selected genotypes. Weight in water is directly associated with relative density. The data are from a single harvest at ten months after planting. In both plots, cooking time is indicated in brackets for each genotype.

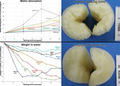

## Introduction

Cassava (*Manihot esculenta* Crantz) is the most widely grown tropical root and tuber crop (Meireles da Silva *et al*., 2003; Parmar *et al*., 2017), and is consumed in virtually all tropical regions. Boiling cassava roots is one of the simplest modes of consumption, common in Latin American and African countries, in particular Paraguay, Colombia, Costa Rica, Haiti, Democratic Republic of Congo, Ghana, Cameroon and Benin. In Asia, boiled cassava is popular in India, Sri Lanka and Indonesia. Cassava cultivars that farmers and consumers select for boiling share specific cooking quality criteria (Bechoff *et al*., 2017), among which softness, *mealiness* (or friability, i.e. the ability to disintegrate between fingers and in the mouth) and short boiling time are most important (Lorenzi, 1994; Hongbété *et al*., 2011).

Consumers by far prefer varieties that cook quickly and fracture or disintegrate during cooking, making the mealiness and texture of boiled roots a very high priority trait for breeders. Cooking quality of boiled cassava is multi‐dimensional (texture, boiling time), and the result of complex processes that depend on several parameters (Eggleston & Asiedu, 1994; Padonou *et al*., 2005; Favaro *et al*., 2008). Dry matter content (DMC) plays a role (Padonou *et al*., 2005; Hongbété *et al*., 2011), as cultivars with DMC levels below 25% tend to have poor cooking quality (Wheatley & Gomez, 1985). However, within the most common range of DMC (32–40%), there is no clear evidence of a relationship between DMC and cooking quality. Both mealiness and boiling time are key but uncorrelated requirements (Ngeve, 2003). Mealiness is related to firmness (Padonou *et al*., 2005), a reduction in cell cohesiveness (Safo‐Kantanka & Owusu‐Nipah, 1992) and water absorption during boiling (Butarelo *et al*., 2004; Favaro *et al*., 2008; Kouadio *et al*., 2011). At the molecular level, pectins and cell wall characteristics have been linked to mealiness (Menoli & Beléia, 2007; Favaro *et al*., 2008; Hongbété *et al*., 2011).

Texture after boiling is another important parameter defining cooking quality in cassava (Wheatley & Gomez, 1985; Safo‐Kantanka & Owusu‐Nipah, 1992; Eggleston & Asiedu, 1994; Hongbété *et al*., 2011; Talma *et al*., 2013). Texture has been assessed by different tools ranging from the simple but arbitrary ‘fork’ method through precise but time‐consuming and labour‐intensive penetrometers. User‐friendly, inexpensive and high‐throughput varietal screening methods for textural characterisations of boiled roots are currently under development by the analytical laboratory team at CIAT and will be the subject of a separate publication.

Large variation in the time required for roots to get soft in response to boiling has been reported and linked to genetic and environmental factors (Pereira *et al*., 1985; Fukuda & Borges, 1988; Borges *et al*., 2002; Beleia *et al*., 2004; Azevedo Miranda *et al*., 2008; Sajeev *et al*., 2010; Talma *et al*., 2013; Iragaba *et al*., 2019). Cooking time (CT) tends to be shorter in plants harvested at a younger age (Lorenzi, 1994; Ngeve, 2003; Chirif, 2013). Calcium (Ca^2+^), magnesium (Mg^2+^) and phytic acid seem to influence CT as well (Eggleston & Asiedu, 1994; Favaro *et al*., 2008; Maieves *et al*., 2012). Larger starch granules may favour mealiness (Safo‐Kantanka & Owusu‐Nipah, 1992) but amylose content does not seem to play a role in cooking quality (Padonou *et al*., 2005).

Routine assessment of cooking quality, including CT, should be performed in breeding for varieties targeting the market for boiled cassava (Dixon *et al*., 1994; Iragaba *et al*., 2019). This is a requirement to increase the likelihood of adoption of improved genotypes. However, the diversity of factors affecting cooking quality and time complicate matters considerably. In particular, current methods to assess cooking quality are time‐consuming. Developing medium or high‐throughput methods would be useful to evaluate large numbers of candidate clones, and fully integrate cooking quality among selection criteria used in cassava breeding. In the current study, 36 cassava genotypes, planted on the same date, were harvested monthly from 8 to 11 months after planting (MAP) and evaluated to understand the relationship among dry matter content, water absorption (WAB), changes related to root relative density (DEN) during boiling (more precisely DEN in this study refers to changes in weight of roots immersed in water) and CT. The objectives were as follows: (i) to identify genotypes with short CT, for variety development; (ii) to explore the effect of plant age on CT; (iii) to investigate whether CT, a key criteria to judge cooking quality, can be predicted by other, more agile parameters including DMC, WAB and change in DEN during boiling; and (iv) to develop faster phenotyping methods for the selection of cassava cultivars requiring short cooking time. Cooking is the standard term used in the literature to describe boiling of cassava roots. This may lead to confusion because there are many ways to cook the roots (boiling, baking and frying being the most common). Cooking and boiling will be considered synonymous terms in the present manuscript.

## Materials and methods

Thirty‐six cassava genotypes with contrasting cooking quality (from short to long CT) were selected from CIAT’s Cassava Germplasm Collection and the breeding program. These genotypes were planted as one replication in 40‐plant plots with completely randomised design in Palmira (1000 m.a.s.l), Colombia, in February 2019. The space between plants within a row and among rows was 1 m. Standard field management and fertilisation was followed (Cadavid‐L., 2012). Irrigation was provided when necessary. The genotypes were harvested monthly (thus at different ages), starting at eight MAP in October 2019, until eleven MAP in January 2020. The four harvests were labelled H‐8M, H‐9M, H‐10M and H‐11M, respectively. Three plants per genotype were harvested each time, to provide enough commercial‐size roots to carry out the experimental work. Two approaches were taken to assess either WAB (Experiment 1) or changes in DEN (Experiment 2) during boiling.

### Experiment 1: Water absorption and cooking time

#### Preparation of root samples for boiling

**Figure 1:**
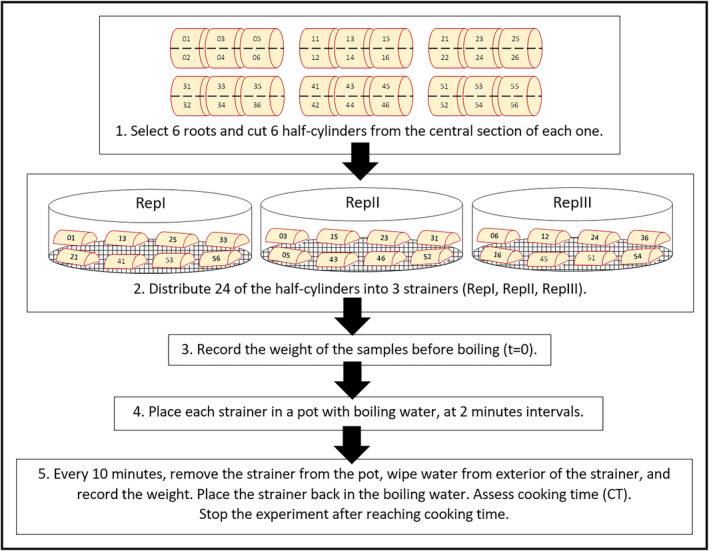
Flow chart of the experimental protocol for water absorption and cooking time (Experiment 1).

For each cassava genotype, six roots (at least 25 cm long and 5.5 cm in diameter at the central part) were selected. Initially, two plants were harvested, and equal number of roots from each of them were taken. If necessary, a third plant was harvested to complete the required number of roots. For each root, both extremes were discarded and six half‐cylinders (about 6 cm long and 5.5 cm diameter) were obtained from the middle section. The pieces were identified individually by numbering the half‐cylinders 01–06; 11–16; 21–26; 31–36; 41–46; and 51–56 from roots 1, 2, 3, 4, 5 and 6, respectively (Fig. 1).

Twenty‐four of the resulting 36 pieces were distributed among three metal strainers (test sieves mesh size 1.4mm, Fisher Scientific Co., Portsmouth, USA), making sure that each strainer contained an even mix of pieces from the six roots as follows: Replication I (RepI): 01, 13, 25, 33, 21, 41, 53 and 56; Replication II (RepII): 03, 15, 23, 31, 05, 43, 52 and 46; and Replication III (RepIII): 06, 12, 24, 36, 16, 45, 51 and 54. The 12 remaining pieces were kept for DMC measurement and further analyses.

#### Boiling of roots and measurement of water absorption and cooking time

The protocols for water absorption and cooking time were adapted, respectively, from Kouadio *et al*. (2011) and Wheatley & Gomez (1985), with modifications (Fig. 1). The three strainers were weighed before boiling (time t = 0). The RepI strainer was placed in a cooking pot with water already boiling. The second strainer (RepII) was placed in a second pot with boiling water two minutes later. Similarly, the third strainer (RepIII) was placed in a third pot with boiling water another two minutes later. The pots contained a quantity of water significantly larger (4 L per 400–600 g roots) than the weight of the roots and their strainer, so as to minimise the reduction in temperature upon introduction of the roots in the water.

When RepI reached ten minutes in boiling water, the strainer was removed from the cooking pot, and the bottom of the strainer was dried with paper towel during 30 s, then weighed with a 0.01 g precision digital scale (Sartorius AX6202, Göttingen, Germany) and immediately placed back in the boiling water. The time the strainer and roots spent outside the boiling water was measured with a timer and recorded (on average, 1 min ± 10 s). The same procedure was followed for RepII and RepIII. The strainers were weighed in the same way every ten minutes until 30 min or until reaching CT in roots requiring longer than 30 min to reach CT (the protocol to evaluate CT is described in the following paragraph). At that point, the experiment was stopped and the strainers removed from boiling water. The change in weight of the root pieces during boiling was calculated as percentage of their initial weight and interpreted as absorption of water (WAB) by the root pieces at various times (t = 0, t = 10, t = 20, t = 30 min, up to t = CT). The quantity of material released by the roots into the cooking water was considered negligible, although this may be accurate only for cooking times until 30 min as preliminary tests indicate that roots from genotypes with short cooking times start to crumble after boiling more than 30 min, thus starting to release significant amounts of material into the water. One source of inaccuracy of the WAB protocol was the convection of hot air above the balance during weighing the sample + strainer. Preliminary tests showed that the display of the balance varied over a maximum range of 20 g due to hot air convection, which given the initial weight of the sample + strainer (ranging from 800 to 1100 g), corresponded to a loss of accuracy between 1.8% and 2.5%.

Cooking time was determined by a trained operator as follows (Wheatley & Gomez, 1985), for all the genotypes included in Experiment 1 (Fig. 1): the pieces of roots were monitored during boiling by carefully probing their surface with a fork and visually assessing their appearance, until becoming soft. The time at which six out of the eight pieces contained in one strainer became soft was recorded as CT of the strainer, after subtracting the cumulated time the strainer was outside boiling water for weighing. The average CT for the three replications was recorded as CT of the respective cassava genotype. This CT protocol is semi‐empirical and relies on the operator, with a precision between 1 and 2 min, and an accuracy of ±5 min.

The above was the optimised protocol for measuring WAB and CT, and applied for the third and fourth harvests (H‐10M and H‐11M). For the first and second harvests (H‐8M and H‐9M), we used a slightly different protocol. Rather than eight root pieces per replication, one root piece per replication was used. However, four and six replications were used for the H‐8M and H‐9M harvests, respectively. For these harvests, the root weight measurement stopped when the roots reached CT, even for genotypes reaching CT before 30 min.

#### Dry matter content (DMC)

Six of the 12 remaining fresh pieces (from six different roots) were homogenised into a uniform paste with a food processor Essen Skymsen Model PA‐7LE (Bateas, Brazil) with a stainless‐steel disc (diameter 210 mm) perforated over 50% of its surface with 3 mm holes and rotating at 438 rpm. The DMC of the resulting mash was determined in duplicate by drying 11 to 14 g at 105 °C for 24 h. Dry matter was expressed as the percentage of dry weight relative to fresh weight (Sánchez *et al*., 2013).

#### Statistical analyses

Data analysis was performed using R (R Core Team, 2019). The genetic and residual variance of the sampling methods for different harvests was calculated using the linear mixed model with replications as the fixed effect and genotype as the random effect (Table 1). The lmer function in R package *lme4* was used to run the linear mixed model. Broad‐sense heritability (Falconer & Mackay, 1998) was calculated using the function H^2^ = Vg/(Vg + Ve) as suggested by Falconer and Mackay in 1998 (see footnote in Table 1). Since roots from one or two genotypes rotted, causing missing data, the best linear unbiased prediction (BLUP) value of DMC and CT was calculated using a linear mixed model, where genotype, harvest time and the interaction between genotype and harvest time were considered as random effects.

**Table 1:** The genetic and residual variances of different sampling methods to assess cooking time in roots from 36 cassava genotypes.

Harvest	Age at harvest (MAP)	Number of reps	Root pieces per rep	Water absorption, 20min			Cooking time (min)		
				V_g_	V_e_	H^2^	V_g_	V_e_	H^2^
H‐8M	8	4	1	24.86	4.86	0.84	158.38	17.34	0.90
H‐9M	9	6	1	10.78	6.13	0.64	63.4	11.66	0.84
H‐10M	10	3	8	33.76	0.70	0.98	184.28	2.82	0.98
H‐11M	11	3	8	12.61	1.19	0.91	138.39	0.03	1.00

V_g_, Genetic variance; V_e_, residual variance caused by the variation among root pieces within the same genotypes and the variation of operation among samples. H^2^ = V_g_/(V_g_ + V_e_), showing the efficiency in controlling experimental errors. MAP, months after planting.

The box plot in ggplot2 was used to visualise the differences among genotypes and harvests in CT and DMC. Multiple comparison was performed using the function LSD.test in R package agricolae with the p value of 0.05 and Bonferroni for adjusting *P*‐values. The R package corrplot was used to visualise the correlation among DMC, CT and WAB. The *P* value for significant correlation was set as 0.05.

### Experiment 2: Assessing changes in root density during boiling

#### Preparation of root samples for boiling

Using the samples harvested in H‐10M, we tested another method to predict CT. For each genotype, 3–5 cassava roots of commercial size were harvested from 1 to 2 plants as one replication. A 10 cm section of the central part of each root was taken. Each section was then peeled and cut into four longitudinal quarters, which is the way roots are usually boiled. The pieces (12–20 per genotype) were then pooled together into a mesh bag and hung from a 0.01 g precision digital scale (Ohaus SP2001 AM Scout Pro, Parsippany, NJ, USA). The initial weight of the pieces in the air was between 1000 and 1100 g.

#### Boiling and weighing samples

The protocol described in this section originates in the hydrostatic method of estimating DMC in different root and tuber crops based on Archimedes' principle. Typically, roots are weighed (‘*weight in air’*) and then submerged in water and weighed again (‘*weight in water’*). The ratio of root density/water density is known as relative density (or specific gravity) and does not require measuring volumes of the root nor of the water. Relative density of starch is above 1.5 (Gevaudan *et al*., 1989; Dey & Harborne, 1990), and on average, cassava roots have about 33.6% DMC (Sánchez *et al*., 2009). Kawano *et al*. (1987) showed a high positive correlation between root‐specific gravity and DMC, and the method is widely used by breeders of root and tuber crops for rapid screening.

During boiling, the starch gelatinises and the granules swell by absorbing water, leading to an increase in volume and a decrease in density. Their relative density – and consequently *weight in water –* should gradually decrease also. In our study, the raw samples were placed in boiling water and the initial weight in water was recorded (time 0) using the (hanging) digital scale. Subsequently, the weight of the root sections that remained immersed in water was recorded every 5 min during boiling. Roots were not removed from the water at any time and boiling lasted for 60 min. Each sample, therefore, provided 13 weight‐in‐water data points through the boiling period. The hydrostatic method was used here to assess changes in relative density (DEN), through the periodic weighing of roots immersed in boiling water. Our hypothesis was that changes of *weight in water* can be used as a proxy reflecting changes in DEN, which are related to water absorption and CT.

#### Statistical analyses

Since there were slight variations in the initial total weight of roots, changes of weight in water through time were expressed as percentage of the initial weight. For each sample, the evolution of weight was depicted as curves starting at time 0 (by default, 100%). As roots boiled, weight in water gradually went down due to WAB and increase in volume (Jarvis *et al*., 1992), resulting in an overall decrease in DEN. Changes in DEN were assessed through three different approaches:

- Area under the weight change curve (AUWCC). The area under the curve depicting changes in weight were estimated using standard geometry formulas. Samples with little change in DEN would be close to 100% of the plot area. Samples with fast and drastic changes in DEN will cover just a proportion of the total area of the plot (e.g. 50%).
- Linear regression coefficient (LINEAR). A straight‐line regression analysis was done for each sample using Proc Reg procedure from SAS. The dependent variable was the weight in water while the independent variable was the respective time of boiling (in minutes)
- First coefficient in a quadratic regression model (QUADRATIC). A quadratic regression analysis was made for each root sample using Proc Reg procedure from SAS. The dependent variable was, as in the previous case, the weight in water. Two independent parameters were used: time of boiling and the square of boiling time.

The three approaches described above are different ways to assess changes in DEN through boiling. The main purpose was to relate changes in DEN with the softening of the roots and CT. Thus, the final steps were regression analyses in which the dependent variable (CT) was linked to one of the three parameters describing changes in DEN (independent variable). CT was measured through the process of boiling the roots in Experiment 2. But, since information from the same root samples harvested the same day was available from Experiment 1, the dependent variable in this last regression analysis was the CT average, from the two experiments, for each of the 34 genotypes.

## Results and discussion

### Experiment 1

As the boiling protocol was under development, the experiment procedure was modified from one harvest to another. The major modification was increasing the number of root pieces used for evaluating WAB and CT. In the first two harvests (H‐8M and H‐9M), WAB and CT were measured piece by piece. In the last two harvests (H‐10M and H‐11M), eight root pieces were measured as one sample, namely one of three replications. For WAB, the modified procedure dramatically reduced the residual variance. The broad‐sense heritability (H^2^) increased from 0.64 (H‐9M) to 0.91 (H‐11M), which indicated that the control of experimental errors improved considerably (Table 1). Similarly, the control of residual variance for CT was also improved (H^2^ from 0.84 in H‐9M to 1.00 in H‐11M; Table 1).

**Figure 2:**
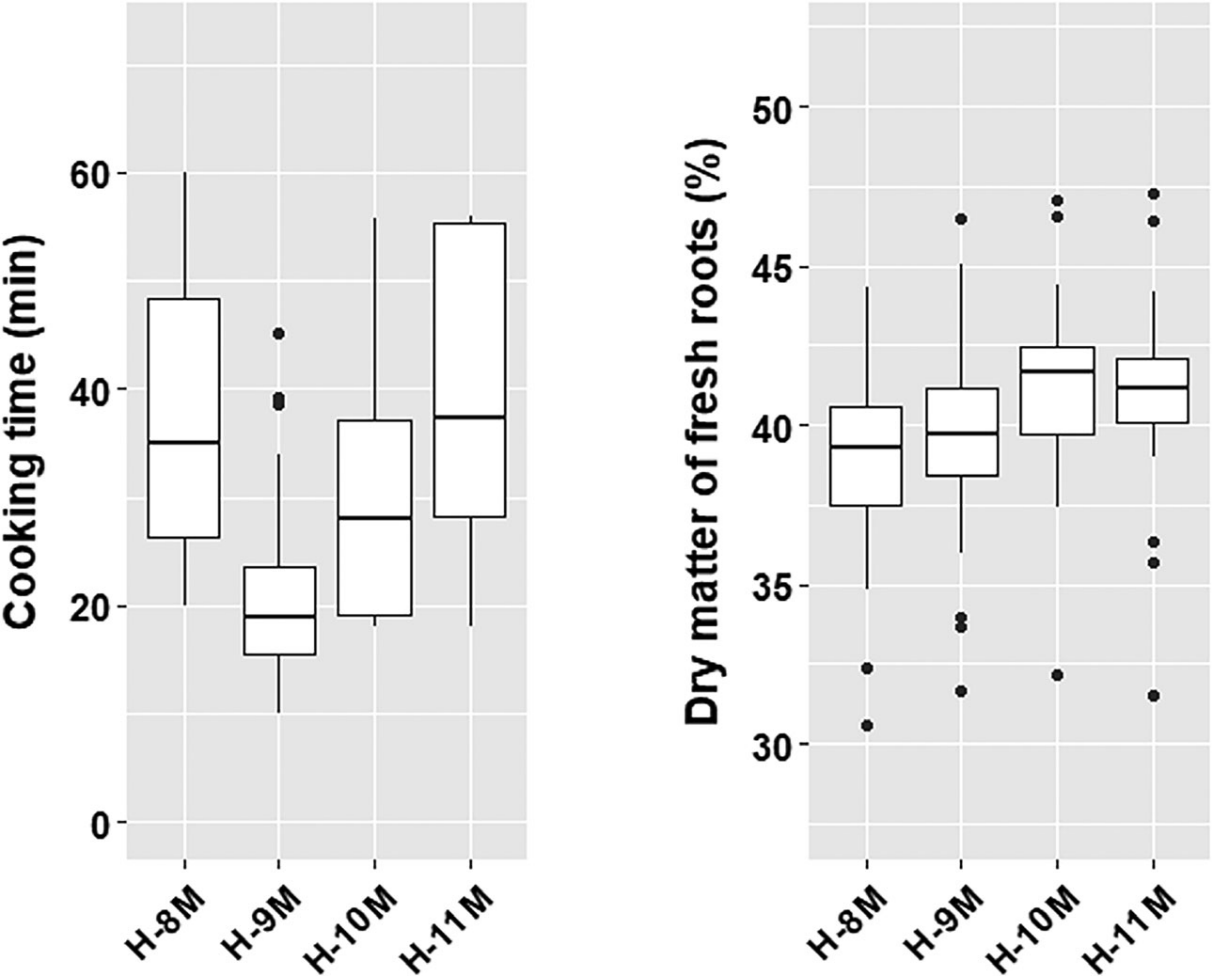
The effect of harvest time on cooking time (CT) and dry matter content (DMC). Significant difference (*P* < 0.05) in CT was observed among harvest time, but not in DMC.

**Figure 3:**
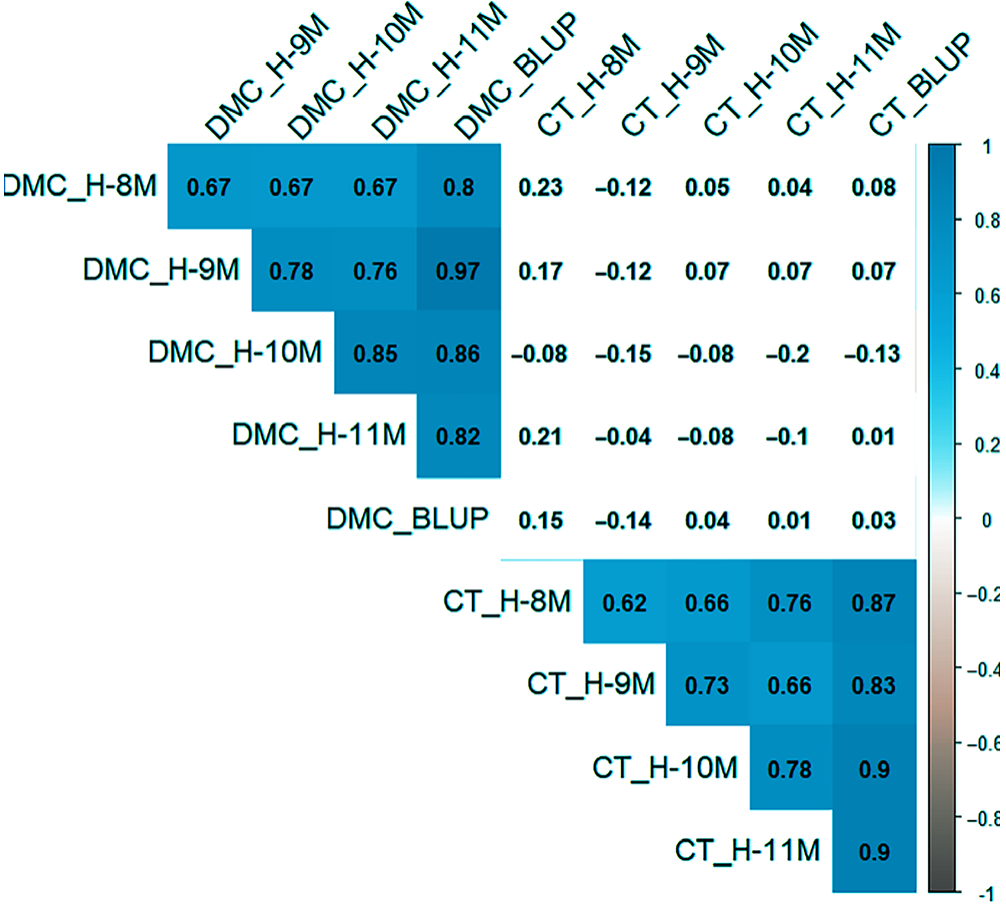
Correlation coefficients (*r*) between cooking time (CT) and dry matter content (DMC), for each separate harvest. CT data were derived from root samples harvested 8, 9, 10 and 11 MAP (H‐8M, H‐9M, H‐10M and H‐11M, respectively). DMC data were derived from root samples harvested 9, 10 and 11 MAP. BLUP values of CT and DMC were calculated using the R package, lme4. Non‐significant correlations (*P* ≥ 0.05) are shown with blank background. Significance and magnitude of significant correlations are shown with varying background colour.

Cassava genotypes showed significant differences in CT between harvests (Fig. 2; *P* ≤ 0.05). In H‐9M, 90% of genotypes required no more than 30 min to reach CT, whereas in H‐8M and H‐11M, more than 60% of genotypes required more than 30 min of boiling. The DMC of fresh roots also changed between harvests (*P* ≤ 0.05). The lowest DMC was observed in H‐9M (9 MAP), while there was no significant difference in DMC between H‐10M and H‐11M harvests (Fig. 2). Although significant differences were observed, CT and DMC still showed significant correlation among harvests, *r* ≥ 0.76 for DMC and *r* ≥ 0.62 for CT (Fig. 3).

**Figure 4:**
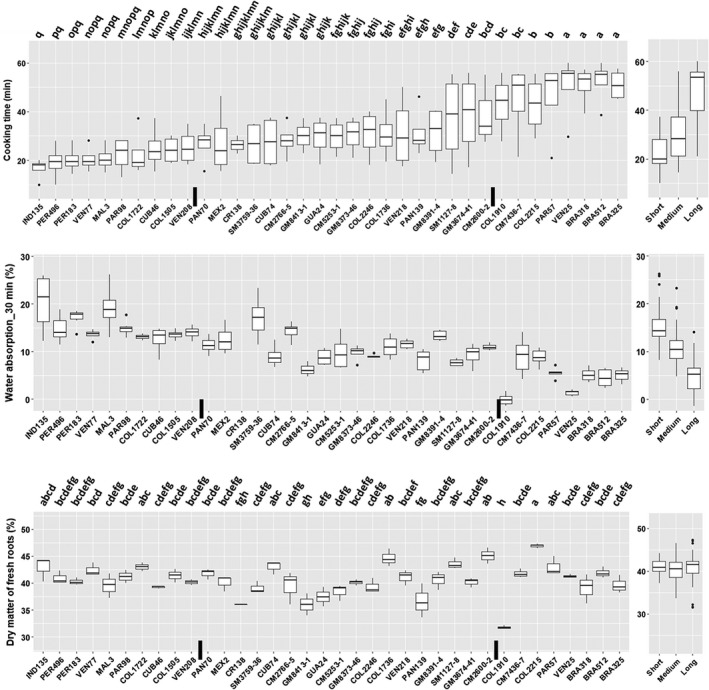
Variation of 36 cassava genotypes in cooking time (CT), water absorption (WAB) and dry matter content (DMC). CT and DMC data were derived from the root samples harvested in 8, 9, 10 and 11 months after planting (MAP). WAB data were from the samples harvested in 10 and 11 MAP. Based on the mean value of CT, the genotypes were divided into three groups, short (CTmean ≤ 25 min), medium (25 min < CTmean ≤ 40 min) and long (CTmean > 40 min). The black vertical bars on the x‐axis show the borders of the groups. The genotypes labelled by different letters or letter combinations are based on multiple statistical comparisons. No statistical significance exists between genotypes labelled by one or more identical letters (*P* ≤ 0.05).

Significant differences (*P* ≤ 0.01) were also observed among the 36 cassava genotypes in terms of CT and DMC (Fig. 4). Genotype IND135 boiled fastest (<20 min) regardless of harvesting time. Six additional genotypes, PER183, PER496, VEN77, MAL3, PAR98 and COL1722, boiled consistently fast in the four harvests (<30 min; Fig. 4). On the other end, five genotypes (PAR57, VEN25, BRA318, BRA512 and BRA325) consistently required more than 40 or 50 min to reach CT. Based on the mean values of CT across harvests, the genotypes were divided into three groups, short (CTmean ≤ 25 min), medium (25 min < CTmean ≤ 40 min) and long (CTmean > 40 min) (Fig. 4).

Unexpectedly, a popular cooking variety at the north coast of Colombia, COL2215, cooked slowly in the present study (CT between 30 and 55 min). COL2215 had the highest DMC among the 36 genotypes (~47%); however, another slow‐cooking genotype, COL1910, showed the lowest DMC (~32%; Fig. 4). There was no significant correlation between CT and DMC (*r* ≤ 0.21; Fig. 3); however, the genotypes with short CT tended to be associated with medium or high DMC (40%–44%, Fig. 4).

**Figure 5:**
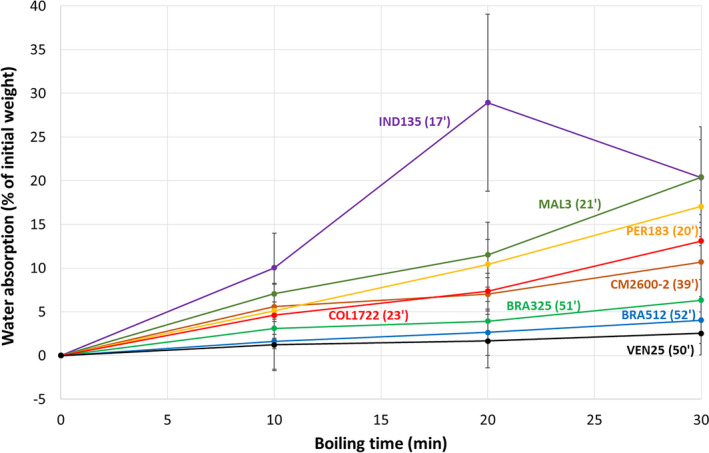
Water absorption during boiling expressed as percentage of the initial weight of root samples from selected genotypes. CT is indicated in brackets for each genotype. The data are averages of the four harvests (H‐8M, H‐9M, H‐10M and H‐11M).

For all genotypes, WAB increased with boiling time (Fig. 5 shows selected genotypes with contrasting behaviour). However, there were significant differences in the magnitude of WAB among genotypes, ranging from less than 5% to 20% of their initial weight after 30 min boiling (Fig. 4). This genetic difference is relevant because high WAB could be associated with short CT. The three groups of genotypes by CT (short, medium and long) (Fig. 4) had average WAB at 30 min of 15.3 ± 3.1, 10.7 ± 1.7 and 4.9 ± 3.8% of initial root weight, respectively. IND135 absorbed markedly more water than other genotypes, with more than 25% WAB in less than 20 min. The decrease in WAB at 30 min observed for IND135 (Fig. 5) was an artefact due to the fragmentation of the pieces of roots into the boiling water.

**Figure 6:**
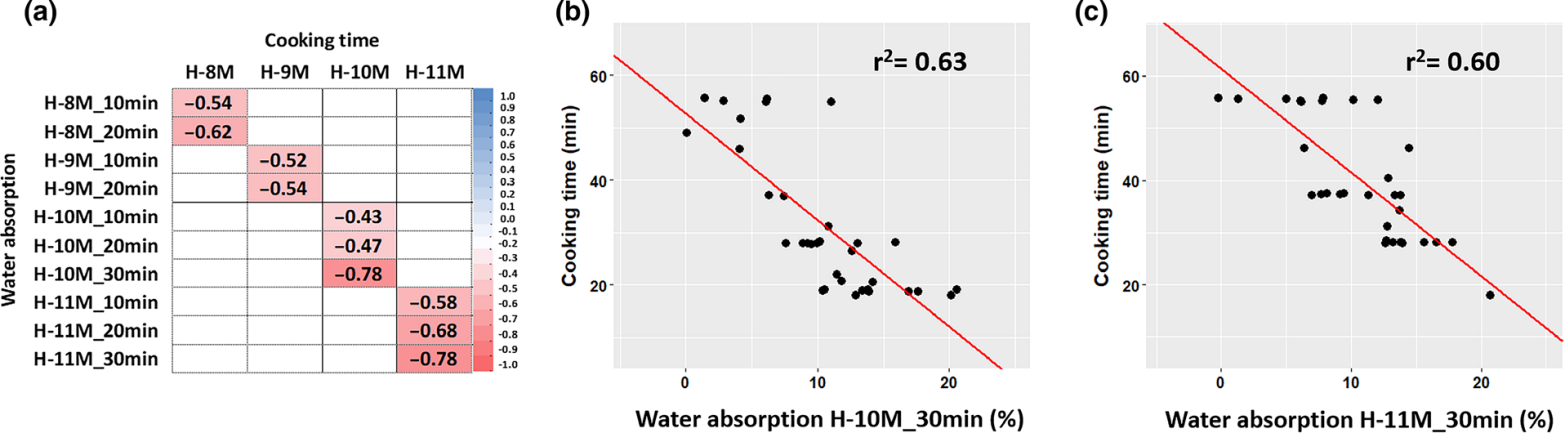
Correlation coefficients (*r*) between water absorption (WAB) and cooking time (CT) in each harvest (a). In H‐8M and H‐9M, the WAB at t = 10 and t = 20 min were recorded, while in H‐10M and H‐11M, the WAB at t = 10, t = 20 and t = 30 min were recorded. Scatter plots show the correlation between CT and WAB at t = 30 min in H‐10M (b) and H‐11M (c).

For each harvest and across genotypes (H‐8M, H‐9M, H‐10M and H‐11M), we observed significant negative correlations between CT and WAB at t = 10 min and t = 20 min (−0.68 ≤ *r* ≤ −0.43; Fig. 6a), but the correlation coefficients were lower than expected and there was a high residual variance, in particular in the case of H‐8M and H‐9M harvests. For the H‐10M and H‐11M, we modified the protocol by (i) pooling eight root pieces as a sample to reduce variance and (ii) measuring WAB for all samples until at least 30 min, even for genotypes reaching CT before 30 min. These changes resulted in better correlations between CT and WAB at t = 30 min (*r* = −0.78, Fig. 6a). Thus, WAB after 30 min boiling explained ~60% of the variance in CT of the 36 cassava genotypes (Fig 6b and c). This result enables a rapid sorting between short‐ and long‐boiling genotypes, if not an accurate prediction of CT, using WAB at t = 30 min data.

**Figure 7:**
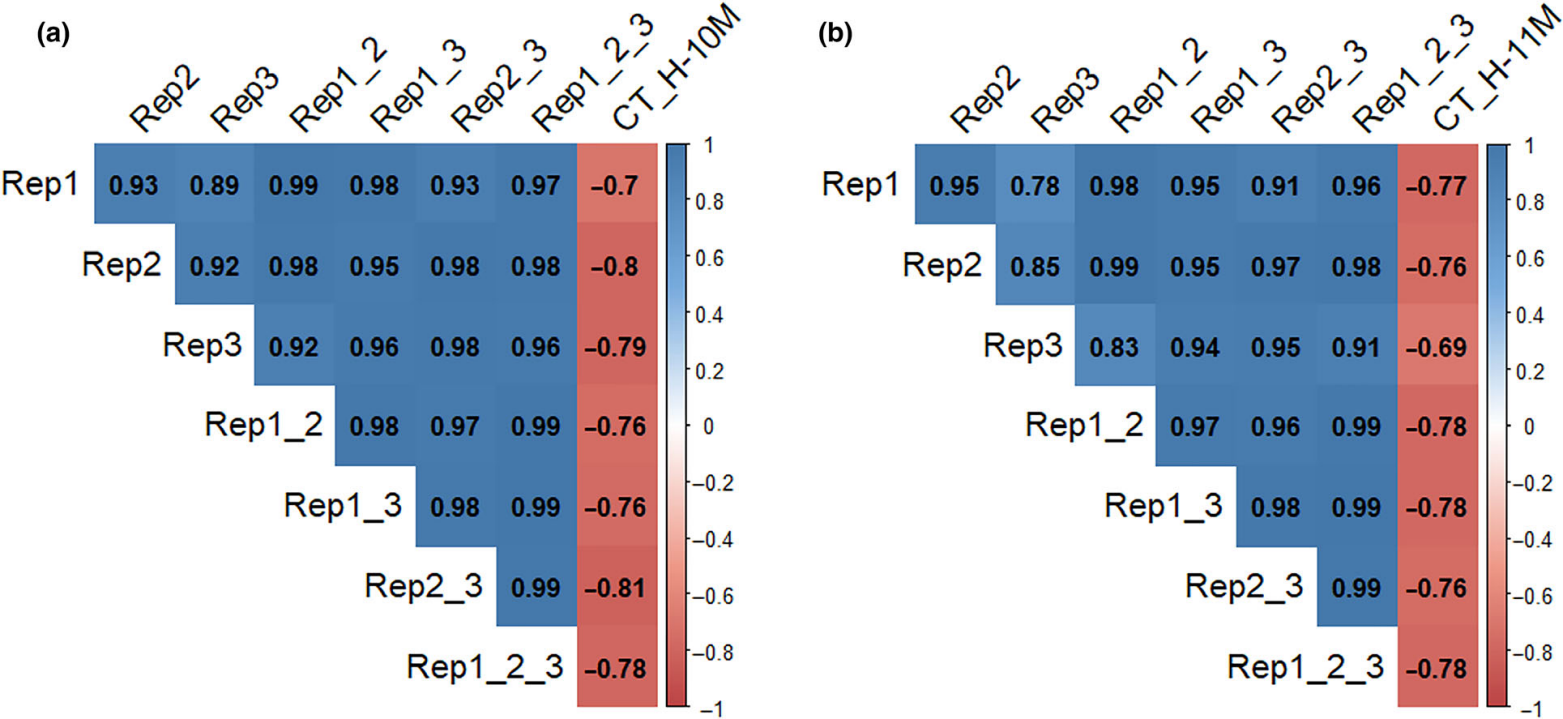
Correlation coefficients (*r*) between cooking time (CT) and water absorption (WAB) at t = 30 min boiling from one, two and three replications. The data were from the H‐10M (a) and H‐11M (b) harvests. Rep1, Rep2 and Rep3 are the WAB at t = 30 min of each separate replication. Rep1_2, Rep1_3 and Rep2_3 are the mean WAB at t = 30 min of replications 1 and 2; 1 and 3; and 2 and 3, respectively. Rep1_2_3 is the mean WAB at t = 30 min of the three replications.

To obtain high‐quality data, we optimised the boiling protocol by using three replications with eight root pieces each. Minimising the number of replications while maintaining data quality is critical for screening large breeding populations. The correlations between CT and mean WAB at t = 30 min from one, two or three replications (Fig. 7) indicated that the means of two replications provided similar correlation coefficients as the mean of three replications (0.76–0.81). Single replications provided slightly lower correlation coefficients (0.69–0.80). Hence, the WAB protocol may be reduced to two replications without affecting data quality, when screening large numbers of genotypes.

Along with appearance, mealiness and flavour, CT is one of the major components of cooking quality in cassava. These are quantitative traits determined by multiple genetic and environmental factors (Eggleston & Asiedu, 1994; Lorenzi, 1994; Ngeve, 2003; Padonou *et al*., 2005; Favaro *et al*., 2008). The current study confirmed this, with significant differences in CT found among genotypes and among harvests. Genotypes with consistently short CT across harvests were identified, in particular IND135, PER183 and VEN77. Similarly, VEN25, BRA318, BRA512 and BRA325 consistently required long boiling times. While significant differences in CT were observed between harvests or plant age, CTs among all harvests were significantly correlated, (*r* ≥ 0.62, Fig. 3).

The correlation coefficient was considerably higher (*r* = 0.78) when the improved protocol was analysed separately (H‐10M and H‐11M harvests). Thus, even though no specific CT threshold could be defined to cut‐off between fast‐ and slow‐cooking genotypes, ranking genotypes by CT was still possible. Selections and rejections based on one harvest, therefore, appropriately reflected other harvests in this study. When breeding for cooking quality, selection based on short CT needs to take this into account in order to address the environmental effects. For example, 30min CT was a long cooking time in the H‐9M harvest, but was considered a short CT in H‐8M and H‐11M harvests (Fig. 2).

### Experiment 2

**Figure 8:**
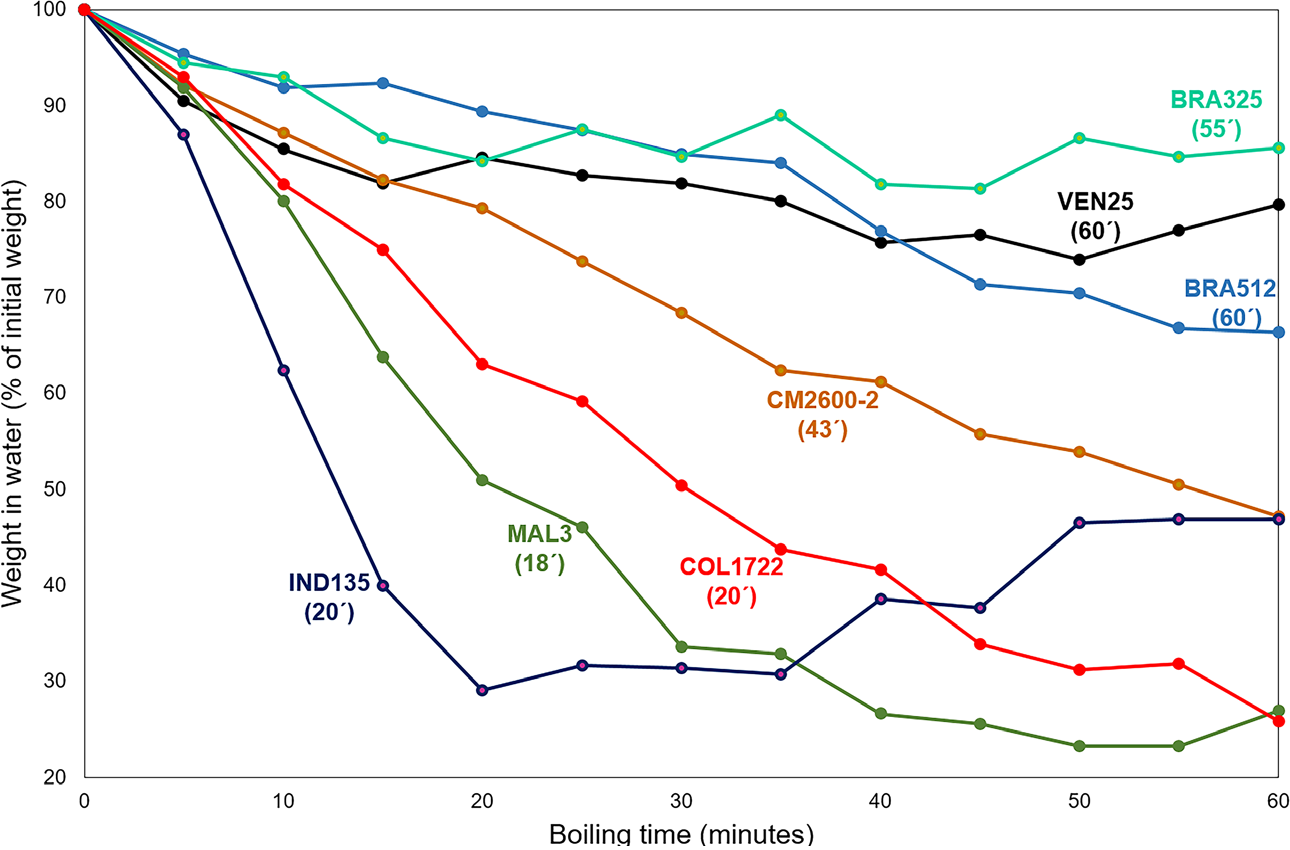
Change in weight in water (expressed as percentage of the weight at time t = 0) during boiling roots from selected genotypes. Cooking time (CT) is indicated in brackets for each genotype. The data are from the H‐10M harvest.

Root samples from CR138 and GM8413‐1 were not available for the change in DEN study which was therefore based on 34 genotypes. Figure 8 presents the change in weight curves from root samples of selected genotypes. The selection of few genotypes was done for clarity and represents extremes in the type of variation observed. BRA325, BRA512 and VEN25 changed little in DEN, and their curves are at the top of the plot. CT for these three genotypes was >55 min. Roots from CM2600‐2 showed an intermediate behaviour. Average CT for this clone was 43 min and DEN decreased faster than those from the previous genotypes and at a rather constant rate (straight negative slope). Roots from COL1722 also lost DEN at more or less a constant rate but faster than CM2600‐2, and CT was also shorter (20 min). Roots from IND135 and MAL3 boiled quickly (CT 20 and 18 min, respectively). The curves for these last two genotypes showed a rapid loss of DEN during the first 20–30 min, and then, the rate change slowed down (a ‘*quadratic response’*). In the case of IND135, weight in water of the roots began to increase at the end of the boiling period in agreement with Fig. 5.

Regression analyses were carried out linking the three parameters assessing change in DEN (AUWCC, LINEAR and QUADRATIC) with CT. In addition to the full data set (boiling through 60 min), regression analyses were also conducted taking data through 50, 40, 30 and 20 min of boiling. Table 2 presents a summary of the different regressions using the three parameters. Experiment 2 aimed at identifying an efficient protocol for predicting CT through the three parameters. Ideally, roots would not need to be boiled for 60 min for predicting CT.

**Table 2:** Coefficient of determination (*r* ^2^) of regression analyses done with data from 20 to 60 min of boiling roots.

Boiling time (min)	Parameter assessing changes in root density		
	AUWCC	LINEAR	QUADRATIC
20	0.44	0.55	0.14
30	0.54	**0.66**	0.34
40	0.59	0.62	0.51
50	0.60	0.52	0.57
60	0.56	0.32	0.63

In the regression analyses, the dependent variable was cooking time (CT), for each genotype. The independent variable was either of the three parameters assessing change in root density, through varying durations of the boiling period. The highest r^2^ value has been highlighted in bold font.

Data presented in Table 2 and Fig. 8 indicated that changes in density, through the different boiling periods, were linked to CT. The best prediction for CT was obtained from the linear regression coefficient on a data set including 30 min boiling (*r* ^2^ = 0.66). When data beyond 30 min boiling were used, the straight‐line regression coefficient lost precision. This is likely because in those samples showing a nearly quadratic response, the linear regression coefficient would increasingly underestimate the speed of DEN loss beyond 30 min of boiling. The second‐best model (based on the *r* ^2^ values) was the first coefficient in the quadratic regression analysis and using the full data set (60 min boiling, *r* ^2^ = 0.63). AUWCC was not an efficient parameter predicting CT. It can be concluded that the best approach to predict CT in a large population of genotypes would be to boil roots for 30 min and use the linear regression coefficient assessing changes in DEN through time.

**Figure 9:**
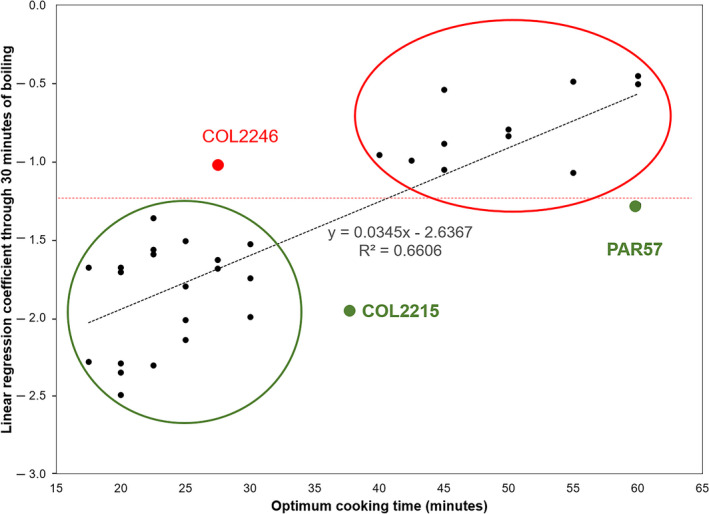
Relationship between cooking time (CT) and the linear regression coefficients between change in root density (through 30 min of boiling, DEN) and CT. With few exceptions, a regression coefficient of −1.25 (horizontal dotted line) splits the samples into those with short (≤ 30 min) and long (>30 min) CT.

The *r* ^2^ value of the best model (0.66) indicates that about two thirds of the variation in CT can be predicted by the linear regression coefficient for roots boiled for 30 min. There is still large variation that remains unaccounted for. Hence, while exact values for CT may not be fully predictable with the LINEAR parameter, a rapid sorting of genotypes between short and long CT would be possible. Indeed, the relationship between CT and the linear regression coefficient through 30 min of boiling (Fig. 9) indicates that genotypes can be split into two such groups (with few exceptions), with a regression coefficient threshold of −1.25. This approach would be useful to pre‐select promising genotypes within the population. The proposed threshold (−1.25) can be fine‐tuned or modified depending on the pros and cons of accepting false positives or rejecting false negatives.

### Advantages of the water absorption and density methods to predict cooking time

No significant relationship between DMC and CT was found in this study, although consumers usually reject varieties with low DMC (<25%). WAB showed significant negative correlations with CT, consistently at 10, 20 and 30 min of boiling. This was particularly true at t = 30 min with the improved protocol (H‐10M and H‐11M harvests), with correlation coefficients *r* = −0.78 in both cases (Fig. 6). These results agree with earlier reports (Butarelo *et al*., 2004; Beleia *et al*., 2004; Kouadio *et al*., 2011). WAB at t = 30 min can therefore be a faster, less subjective and more repeatable method to predict CT for cassava breeding efforts compared to direct measurement. To measure CT directly required up to 60 min for some genotypes. Moreover, as discussed above, due to environmental effects, CT had limited informative value, as no specific threshold defining fast‐cooking could be established. On a practical level, measuring CT directly requires well‐trained personnel and remains a somewhat subjective measurement. WAB at 30 min boiling, on the other hand, provides a simple and objective method to predict CT, requiring only weighing the roots at time 0 and after 30 min boiling, instead of a constant inspection until the roots reach the desired softening (using a fork multiple times). The proposed technology would be convenient as a pre‐selection tool when many segregating progenies need to be screened. Up to 100 samples per day may be managed by one person using the WAB method (not including root washing & peeling, and sample preparation).

The protocol to quantify WAB was improved to minimise the residual variance: (i) at least six commercial roots from three plants were used. Each root was cut into six half‐cylinder pieces, and the root pieces were randomised; (ii) at least eight root pieces from the six roots were put together as one sample for measuring WAB; (iii) though two or three replications are recommended, just one may also provide reliable information for screening large breeding populations. If one replication is used, increasing the number of root pieces (≥12) sampled from several different roots would be advisable.

The correlation between DEN change during boiling and CT (*r* ^2^ up to 0.66) indicated that this variable could be used to predict CT of cassava roots. The main advantage of predicting CT through monitoring changes in DEN is that it can be automated. Also, several data points are recorded during boiling and are eventually calculated as a single value. This makes the technique more robust and less vulnerable to mistakes. Given the limitations of direct CT measurement (discussed above) and the fact that marked variations can be observed even between different sections of the same root (Lorenzi, 1994), the proposed density method could facilitate greatly the screening of large numbers of genotypes. A battery of boilers can be set‐up, each one with its own digital scale connected to a data logging device. A bulked root sample of about 1000 g per genotype (with sections from different roots and different plants) can be boiled for 30 min while the system automatically records weight in water, enabling rapid pre‐selection of promising genotypes. Changes related to WAB (Experiment 1) and DEN (Experiment 2) agree with each other. However, Experiment 2 was conducted only for one (H‐10M) of the four harvesting dates. The coefficient of determination for DEN was slightly higher (0.66) than that for WAB (0.61) for the H‐10M harvest.

## Conclusions

Clear genetic differences for cooking time (CT) among the 36 genotypes evaluated were found. Roots from 15 genotypes cooked ≤25 min; CT in eight clones ranged between 25 and 40 min, whereas the roots from the remaining 11 genotypes required more than 40 min or did not cook at all. Roots from MAL3 and MEX2 cooked in l7.5 min. Varieties COL1722, GM 8391‐4, IND135, PER496 and VEN77 were also outstanding (their roots cooked in 20 min). Dry matter content tended to increase with the age of the plant. Although the average CT varied for each harvesting, there was no clear trend: the lowest CT was observed in roots harvested 9 and 10 MAP and the highest in those harvested 8 and 11 MAP. There was no clear correlation between dry matter content and CT. On the other hand, water absorption and changes in relative density showed significant correlations with CT, particularly when evaluated after 30 min boiling. As expected, water absorption and changes in relative density are clearly associated. We propose two alternative protocols (based on water absorption and changes in relative density) as promising methods to predict CT. They are simple, objective and can achieve medium throughput (up to 100 samples/day/operator). The protocol based on water absorption is more labour‐intensive but provide more information. The method based on changes in relative density offers the advantage of simplicity and the possibility of automatisation, but provides a single response variable. Further tests have been arranged to compare the two methods and confirm the reliability in a wider set of genotypes.

## Author contribution

**Thierry Tran:** Conceptualization (equal); Formal analysis (equal); Writing‐original draft (equal). **Xiaofei Zhang:** Data curation (equal); Formal analysis (equal). **Hernán Ceballos:** Formal analysis (equal); Investigation (equal); Methodology (equal); Writing‐review & editing (equal). **Jhon Larry Moreno:** Investigation (equal); Methodology (equal). **Jorge L Luna:** Investigation (equal); Methodology (equal). **Andrès Escobar:** Investigation (equal); Methodology (equal). **Nelson Morante:** Investigation (equal); Methodology (equal). **John Belalcazar:** Methodology (equal); Supervision (equal). **Luis Augusto Becerra:** Project administration (equal). **Dominique Dufour:** Methodology (equal); Project administration (equal); Resources (equal); Supervision (equal).

## Conflict of interest

The authors declare no conflict of interest.

## Ethical approval

Ethics approval was not required for this research.

### Peer review

The peer review history for this article is available at https://publons.com/publon/10.1111/ijfs.14769.

## Data Availability

Data available on request from the authors.
